# The Medical Care of Idiots, Imbeciles, and Feeble-Minded Children

**Published:** 1895-03-30

**Authors:** G. E. Shuttleworth

**Affiliations:** late Medical Superintendent, Royal Albert Asylum, Lancaster


### The Medical Care of Idiots, Imbeciles, and Feeble-Minded Children.

By G. E. Shuttleworth, B.A., M.D., &c., late Medical Superintendent, Royal Albert Asylum, Lancaster.

It has been pointed out in previous papers on the subject (see The Hospital, March 24th, May 5th, July 21st, Aug. 18th, and Sept. 8th, 1894) that mentally-deficient children are usually defective in body as well as in mind. Idiocy, in fact, must be considered not merely as a mental affection, but as a vice of the entire organism. Physical abnormalities and defects are consequently frequently met with. Arrest of development in intra-uterine life accounts for some of the more striking defects, such as microcephalus (the head circumference in these cases ranging from fifteen to eighteen inches), and the unfinished appearance of imbeciles of the so-called Mongol type. It is also accountable for a variety of nervous defects and disorders proceeding from an imperfectly-formed brain and nervous system. In one case occurring in the experience of the writer, absence of two-thirds of the cerebellum was discovered at the autopsy; in another there was the defect called povencephalus—that is, a gap extending in the place of the right frontal convolutions, and leaving the deeper structures of the brain quite uncovered. In the former case but few characteristic symptoms were observed during life; in the latter there was unilateral paralysis, with spasmodic movements of the fingers and toes (athetosis). The weight of the microcephalic brain is sometimes not more than one-fourth of that normal for the age; thus, in a recent case of a man of 29, who died at the Royal Albert Asylum, the brain weighed only twelve and a-half ounces. In the "Mongol" variety the brain is of fair weight, but its convolutions are extremely coarse, so that the proportion of grey matter is comparatively small. It is not, however, by "naked eye" appearances only that the cerebral defects of idiots must be gauged. Microscopic examination will discover in most instances minute structural abnormalities, such as the preponderance of simply-formed brain cells devoid of processes, denoting persistence of fœtal structures, or, on the other hand, degenerative changes resulting from post-inflammatory atrophy. Of the non-congenital types of idiocy, the lesions of hydrocephalus, sometimes attended with persistent effusion, and the thickened membranes, with starved brain tissues, following meningitis, are most commonly met with. In addition, spinal defects and disorders are constantly associated with idiocy. Oscillating eyeballs (nystagmus) and various muscular spasms and paralyses are very common.

So much for nervous defects and disorders. We often find also striking abnormalities in bodily conformation. Hare-lip and cleft palate, deformities of the mouth and tongue, irregularities of dentition, supernumerary fingers and toes, huge hairy moles, and other tegumentary peculiarities have been noted in various cases; and a type of features more theroid than human, sometimes gives rise to a superficial resemblance to such animals as the ape or the sheep. Constitutional predispositions to degenerative diseases, such as scrofula and rickets, also leave their impress upon the external characters of the body; and it must be remarked that as a rule the growth of the idiot is stunted as compared with that of the normal child of the same age. The deficiency both in height and weight of the former from the normal standard goes on increasing from year to year, and the average defect at twenty years of age has been reckoned at three inches in height and 23½ lbs. in weight.

Considering that probably at least one-third of the imbecile class are subject to epilepsy in greater or less degree, and that (to quote Dr. Ireland) "perhaps two-thirds, or even more, of all idiots are of the scrofulous constitution," we shall expect to find a low standard of health and a high death-rate prevailing amongst them. The death statistics of the idiot institutions enable us to judge of their comparative vitality, and it has been calculated that between the ages of five and twenty the mortality of idiots in institutions is about nine times that of the ordinary population of corresponding age. Phthisis or some other form of tubercular disease is responsible for from 50 to 75 per cent. of this mortality.

It would be impossible in the limits of this paper to do more than give very general hints as to treatment. The inappropriateness of all lowering measures is, of course, obvious. Defects of nutrition must be corrected by a liberal and judicious diet, the administration of cod liver oil and maltine preparations, and of ferruginous tonics, such as Parrish's Chemical Food. The maintenance of bodily warmth must be well looked after, and the tendency to mucous flux and to diarrhœa must be combated by the judicious use of astringent remedies.

The most notable advance in the therapeutics of mental defect is the successful treatment of cases of sporadic cretinism by the administration of thyroid gland. The name of "sporadic cretinism" was applied by Dr. Hilton Fagge in 1871 to that peculiar class of idiotic dwarfs characterised by absence or atrophy of the thyroid gland; but it was not till within the last five years that the introduction of thyroid gland tissue from the lower animals was proposed as a treatment for such subjects. Profiting by the experimental investigations of Schiff, Fuhr, and Victor Horsley, various surgeons and physicians tried in these cases, first thyroid grafting, and subsequently the ingestion either of gland extract or of the gland itself; and when the proper doses and modes of administration had been ascertained the results recorded were most satisfactory. In some cases under the personal observation of the writer at the Royal Albert Asylum (of which a full account has been published by his successor, Dr. Telford-Smith) the improvement occurring from the administration by the mouth of thyroid gland (at first ⅛ gland twice weekly; subsequently a five-grain tabloid daily) was simply marvellous. A boy of nine so treated grew 4¼ inches in nine months, having previously been at a standstill as regards stature, and a corresponding impetus was exerted upon his sluggish mental faculties, transforming him from a dull, inert dwarf, to an active child of considerable vivacity. As in the analogous case of myxœdema, it seems necessary in order to secure lasting benefit to continue the treatment, though in modified doses, *en permanence*.

